# Development of Phenol-Enriched Olive Oil with Phenolic Compounds Extracted from Wastewater Produced by Physical Refining

**DOI:** 10.3390/nu9080916

**Published:** 2017-08-22

**Authors:** Francesca Venturi, Chiara Sanmartin, Isabella Taglieri, Anita Nari, Gianpaolo Andrich, Erika Terzuoli, Sandra Donnini, Cristiano Nicolella, Angela Zinnai

**Affiliations:** 1 Department of Agriculture, Food and Environment, University of Pisa, Via del Borghetto 80, 56124 Pisa, Italy; chiara.sanmartin@unipi.it (C.S.); isabella.taglieri@for.unipi.it (I.T.); anita.nari@agr.unipi.it (A.N.); gianpaolo.andrich@unipi.it (G.A.); angela.zinnai@unipi.it (A.Z.); 2 Interdepartmental Research Center Nutrafood-Nutraceuticals and Food for Health, University of Pisa, Via del Borghetto 80, 56124 Pisa, Italy; 3 Department of Life Science, University of Siena, Via Aldo Moro, 2, 53100 Siena, Italy; terzuoli8@unisi.it (E.T.); sandra.donnini@unisi.it (S.D.); 4 Department of Civil and Industrial Engineering, University of Pisa, Largo Lucio Lazzarino, 56122 Pisa, Italy; cristiano.nicolella@unipi.it; 5 Consorzio Polo Tecnologico Magona, Via Magona, 57023 Cecina (LI), Italy

**Keywords:** refining wastewater, enriched olive oil, antioxidant capacity, phenols, tyrosol, hydroxytyrosol, in vitro model, endothelial cells, cardiovascular diseases, cancer diseases

## Abstract

While in the last few years the use of olive cake and mill wastewater as natural sources of phenolic compounds has been widely considered and several studies have focused on the development of new extraction methods and on the production of functional foods enriched with natural antioxidants, no data has been available on the production of a phenol-enriched refined olive oil with its own phenolic compounds extracted from wastewater produced during physical refining. In this study; we aimed to: (i) verify the effectiveness of a multi-step extraction process to recover the high-added-value phenolic compounds contained in wastewater derived from the preliminary washing degumming step of the physical refining of vegetal oils; (ii) evaluate their potential application for the stabilization of olive oil obtained with refined olive oils; and (iii) evaluate their antioxidant activity in an in vitro model of endothelial cells. The results obtained demonstrate the potential of using the refining wastewater as a source of bioactive compounds to improve the nutraceutical value as well as the antioxidant capacity of commercial olive oils. In the conditions adopted, the phenolic content significantly increased in the prototypes of phenol-enriched olive oils when compared with the control oil.

## 1. Introduction

The Mediterranean diet, where olive oil (OO) is the main source of fat, has been shown to reduce the incidence of age-associated diseases, including cardiovascular diseases, cancer, and neurodegenerative diseases [1]. Olive oil contains many bioactive components apart from oleic acid, including polyphenols. In preclinical and clinical studies, polyphenols have been reported to be responsible for some of the properties of olive oil, including anti-atherogenic, anti-inflammatory, anti-aging, anti-tumor, anti-viral, and immune modulator activities [1,2,3]. In 2011, the European Food Safety Authority (EFSA) endorsed a claim regarding the effectiveness of olive oil polyphenols (5 mg/day) in protecting low-density lipoprotein (LDL) from oxidation [4], resulting in a significant anti-atherogenic effect [5].

According to European Union legislation [6], olive oil is classified into categories reflecting its quality and organoleptic properties, namely extra virgin olive oil (EVOO), virgin olive oil (VOO), lampante virgin olive oil (LVOO), refined olive oil (ROO) and also olive oil (OO), among others [7].

In particular, ROO is a low-quality oil that undergoes chemical or physical intervention to become edible, as it is usually obtained from VOO mechanically extracted from damaged olive fruits or from olives stored in unsuitable conditions and using refining methods. It has free acidity, expressed as oleic acid, of not more than 0.3 g per 100 g of oil [8], and is gaining importance in the food industry [7]. Further, ROO has a very reduced content of polyphenols since these compounds are among the substances eliminated during the refining process [9], so it is therefore unstable and subjected to rapid oxidation during storage [10].

As widely reported in the literature, oil manufacturers aim at producing foods that maintain their shelf life and nutritional quality over a defined period [11,12,13]. Thus, the use of antioxidants to minimize the oxidation of lipids in food materials is extensively accepted [14]. In this context, to overcome the stability problems of oils and fats, synthetic antioxidants such as butylated hydroxyanisole (BHA), butylated hydroxytoluene (BHT), and tert-butylhydroquinone (TBHQ) have been used as food additives [10]. However, recent reports reveal that these compounds may be implicated in many health risks, including cancer and carcinogenesis [15,16]. Due to these safety concerns, there is an increasing trend among food scientists to replace these synthetic antioxidants with natural ones, which are generally supposed to be safer [17,18].

In the last few years, the use of olive cake and mill wastewater as a natural source of phenolic compounds has been widely considered, and several studies have focused on the development of new extraction methods [11,13], as well as on the production of functional foods enriched with natural antioxidants [18,19]. In particular, oil-in-water emulsions formulated with stabilizers and enriched with phenolic compounds extracted from olive mill wastewater have recently been studied for the realization of emulsion-based food products with enhanced health properties [20,21]. However, to the best of our knowledge, no data are available in the literature on the feasibility of the production of a phenol-enriched refined olive oil using its own phenolic compounds extracted from wastewater produced during physical refining. 

In this context, this study had three main objectives: (i) to verify the effectiveness of a multi-step extraction process to recover the high-added-value phenolic compounds potentially contained in wastewater produced during the preliminary washing degumming step of physical refining of vegetal oils; (ii) to evaluate their potential application for the stabilization of olive oil obtained with refined olive oils; and (iii) to evaluate their antioxidant activity in an in vitro model of endothelial cells.

## 2. Materials and Methods

### 2.1. Samples

The control olive oil (COO) was utilized as a matrix to carry out phenolic enrichment, and was obtained by mixing virgin olive oil (VOO, 7%) with refined olive oil (ROO, 93%), produced by a physical refining process at the industrial plant for vegetal oil refining managed by SALOV S.p.A. (Massarosa, Lucca, Italy).

Generally speaking, physical refining (also known as steam refining) is an industrial continuous process of deacidification and refining of crude oils as an alternative to alkali (chemical) neutralization [22]. In the proposed experimental protocol, the phenolic extracts were obtained from the wastewater taken at the physical refining plant outlet after the preliminary water degumming step before the steam distillation.

Both COO and wastewater samples were immediately stored at −20 °C in an inert atmosphere (N_2_) to avoid oxidative damage.

As reported previously in Reference [11], the general chemical parameters (free acidity (% of oleic acid), peroxide value (meq O_2_/kg), and K_270_) of the starting COO as well as of the wastewater and phenol-enriched oil were determined at the Laboratory of Food Technology of DAFE (University of Pisa, Italy) according the analytical methods described in Regulation 2568/1991 of the European Union Commission and later modifications. The chemical composition of the starting COO and wastewater are reported in Table 1.

### 2.2. Reagents

Phenolphthalein 1%, Folin–Ciocalteau reagent, and formaldehyde 40% (*m/v*) were purchased from Titolchimica (Pontecchio Polesine, Italy). Acetic acid, ethanol (99.8%), methanol, sodium carbonate anhydrous, diethyl ether, 2,2,4-trimethylpentane, hexane, hydrochloric acid 37%, sodium hydroxide 0.1 N, sodium thiosulphate 0.01 N, potassium persulfate, potassium iodine, chloroform, starch solution indicator 1%, as well as 2,2′-azinobis(3-ethylbenzothiazoline-6-sulphonic acid) (ABTS), tyrosol, the standard of Trolox, tris(hydroxymethyl)aminomethane hydrochloride (Tris HCl), NaCl, NaF, H_2_O_2_, ethylene glycol-bis(β-aminoethyl ether)-*N*,*N*,*N*′,*N*′-tetraacetic acid (EGTA), and Triton were purchased from Sigma Aldrich (Milan, Italy). Hydroxytyrosol was obtained from Cayman Chemicals, Vinci Biochem (Vinci, Italy). DCFH2-DA (2,7-dichlorodihydrofluorescein diacetate) was purchased from Invitrogen (Milan, Italy). The standard gallic acid was supplied by Carlo Erba (Milan, Italy). Fetal calf serum was from EuroClone SpA (Milan, Italy). Diff-Quik was from Mertz-Dade AG, Dade International, (Milan, Italy), anti-caspase-3, anti-catalase and anti-SOD were from Cell Signaling, (Milan, Italy), secondary antibodies were from Promega (Padova, Italy), and cell culture dish and plates were from Sarstedt (Verona, Italy). 

### 2.3. Preparation of Phenolic Extracts from Wastewater

To maximize the recovery of the phenolic compounds from wastewater collected during physical refining, we studied different extraction processes by utilizing two different solvent solutions: ethanol (99.8%), named sol. A; ethanol:diethyl ether (1:2 *v/v*), named sol. B.

In each extraction run, a sample of 25 mL of wastewater was rotary evaporated (Laborota 4000, Heidolph Instruments GMBH and Co. KG, Schwabach, Germany) until all water had been eliminated (150 rpm; 40 °C); the resulting extract was dissolved in 50 mL of extraction solution, shaken for 15 min, and then centrifuged (10,000 rpm (16,770× *g*), 5 min). The total phenol content of supernatant was determined spectrophotometrically at 280 nm (optical path = 1 mm), and calculations were performed using a calibration curve prepared with gallic acid as standard [23] for both solutions utilized for the extraction.

Finally, each extracted solution was rotary evaporated, and the resulting dry extract was stored at −20 °C under N_2_ until its use in the oil enrichment. In these conditions, it was possible to store dry extracts, avoiding the addition of any antioxidant agent.

### 2.4. Preparation of Phenol-Enriched Oil

COO was used as a matrix enrichment by adding one of the wastewater extracts (WW-A, WW-B) to reach the maximum phenol concentration according to the chemical composition of the COO. Thus, two different phenol-enriched oils prototypes (PE-A and PE-B) were prepared. The extracts—obtained as described below—were incorporated into 25 mL of COO and kept shaking for 3 h in an inert atmosphere (N_2_) in the dark at room temperature (20 ± 1 °C). Finally, the phenol-enriched oil prototypes were collected after centrifugation (IEC CL31R Multispeed, Thermo Scientific, Melegnano, Milan, Italy) at 10,000 rpm (16,770× *g*), 5 min, 15 °C, to eliminate any unsolved residues of dry extracts and maintained at 12 ± 1 °C until analysis.

### 2.5. Preparation of Phenolic Extracts from COO and Phenol-Enriched Oil

For the preparation of phenolic extracts from COO and phenol-enriched oil, we followed the method described by Montedoro et al. [24] with some modifications: liquid–liquid extraction with a solution of methanol:water (80:20 *v/v*) was carried out on the COO and phenol-enriched oil samples obtained as described before. In particular, 10 mL of oil samples were mixed with 10 mL of the mix solution, then the mixture was vigorously shaken for 3 min and, after 3 min of stopping in the dark, centrifuged for 15 min at 4000 rpm (2683× *g*). The phases were separated and the extraction was repeated successively two extra times. All the supernatant solutions obtained were stored at −20 °C under N_2_ atmosphere overnight, until their use for total phenol content determination.

### 2.6. Total Phenol Content Determination (Wastewater, Phenolic Extracts from COO, and Phenol-Enriched Oils)

The total phenol content of the wastewater and phenolic extracts from COO and from phenol-enriched oils were determined colorimetrically at 765 nm, using the Folin–Ciocalteau reagent [25].

The total content of non-flavonoid phenolic compounds was determined according to the Kramling and Singleton method, as previously described [23].

Calculations were performed using a calibration curve prepared with gallic acid as the standard.

### 2.7. Bitter Index (BI)

In all samples (COO, PE-A, and PE-B), the bitter index was determined as reported by Gutierrez et al. [26]. Octadecyl (C_18_) disposable extraction columns (6 mL) from J.T. Baker Chemical Company (Phillipsburg, NJ, USA) were used. For the extraction procedure of the bitter components, a sample of 1.0 ± 0.01 g virgin olive oil was dissolved in 4 mL hexane and passed over the C_18_ column, previously activated with methanol (6 mL) and washed with hexane (6 mL). After elution, 10 mL hexane was passed to eliminate the fat, and then the retained compounds were eluted with methanol:water (1:1) to 25 mL in a tared beaker. The absorbance of the extract was measured at 225 nm against methanol:water (1:1) in a 1 cm cuvette.

### 2.8. Antioxidant Capacity of the Olive Oils (COO and Phenol-Enriched Oils) by ABTS Assay

Antioxidant assay of methanolic extracts was performed following Sgherri et al. [27]. The radical cation ABTS (2,2′-azino-di-[3-ethylbenzthiazoline sulphonate]) was generated as described by Pellegrini et al. [28]. The radical solution was diluted in water to obtain an absorbance at 734 nm of 0.70 ± 0.05. After 5 min from the addition of the extract (1% v/v), the decrease in absorbance was monitored and compared to that of the Trolox standard. The activities of the extracts were quantified by using a dose-response curve of Trolox in the 0.2–1.5 mM range, expressing them in terms of Trolox equivalent antioxidant capacity (TEAC) L^−1^ extract.

### 2.9. Cell Culture

Human umbilical cord vein endothelial cells (HUVEC) were from Cambrex and were maintained in basal Endothelial Growth Medium (EGM-2) and 10% fetal calf serum (FCS, Hyclone). Cells were split 1:3 twice a week, and used until they reached passage seven. 

### 2.10. Cell Growth

The 1.5 × 10^3^ cells resuspended in 10% fetal calf serum (FCS) were seeded in 96-multiwell plates. After adherence, cells were serum starved (0.1% FCS) for 24 h to synchronize cells, and then stimulated with test substances (25, 50, 100, and 200 µM hydrogen peroxide, H_2_O_2_, hydroxytyrosol, (HT), and tyrosol (Tyr), 0.1, 1, 10, 100 µM). After 48 h, cells were fixed in 100% methanol and stained with Diff-Quik. The total cell number/well was counted in a blinded manner at 10× magnification.

### 2.11. Reactive Oxygen Species (ROS) Measurement

Intracellular ROS was evaluated by a fluorimetric method. HUVEC cells (1.5 × 10^3^ cells) were seeded in a 96-multiwell plate, and after adherence, were pre-treated with HT 10 µM and Tyr 10 µM, 30 min or 18 h, and then with H_2_O_2_ (100 µM, 90 min) in a medium without phenol red (for the concentration of HT and Tyr, please see Results Section 3.4). DCFH2-DA was added (10 μM, 30 min) and intracellular levels of ROS were evaluated with a microplate reader (excitation/emission 495/527) (Infinite 200 Pro SpectraFluor). Results are reported as relative fluorescence units (RFU) corrected for the cell number counted.

### 2.12. Western Blot

For Western blot analysis, 3 × 10^5^ cells (HUVEC) were plated in 6 cm diameter dishes. After 24 h, cells were exposed to 1% FCS (Control condition, Ctr) or H_2_O_2_ in the presence or absence of polyphenols (HT 10 µM and Tyr 10 µM). Cells were scraped in a lysis buffer containing 50 mM of Tris HCl (pH 7.4), 150 mM NaCl, 1 mM EGTA, 10 mM NaF, 1% Triton, and 1% protease inhibitor cocktail. Equal amounts (50 μg) of protein were separated by SDS-PAGE onto a gradient 4–12% gel and transferred to a nitrocellulose membrane. The membranes were blocked (1 h) in a solution of 5% (*w/v*) milk and then incubated overnight at 4 °C with the primary antibodies: anti-caspase-3, anti-catalase, and anti-superoxide dismutase (SOD) (each at 1:1000). After 1 h incubation in a secondary antibody anti-IgG horseradish peroxidase (HRP, diluted 1:2500), the immune reaction was revealed by a chemiluminescence system (ChemiDoc, BioRad, Milan, Italy). Results were normalized to those obtained by using an antibody against beta-actin or total caspase-3, when appropriate.

### 2.13. Statistical Analysis

To test the difference between the means among data sets (two replicates for each determination) One-way completely randomized ANOVA (CoStat, Cohort 6 software) was utilized. Comparisons among means were performed by the Bartlett’s X2 corrected test (*p* < 0.05). Tukey’s HSD multiple mean comparison test (*p* < 0.05) was used to state the differences among variables.

For biological analysis, results were either the representative or average of at least three independent experiments done in triplicate. Statistical analysis was performed using ANOVA test and *t*-test for unpaired data (Prism, GraphPad, La Jolla, CA, USA). *p* < 0.05 was considered statistically significant.

## 3. Results and Discussion

### 3.1. Total Phenol Content of Wastewater and Wastewater Extracts

The total phenol content of wastewater as well as of the two phenolic extracts (WW-A and WW-B) obtained are reported in Table 2.

As seen in Table 2, the wastewater collected during olive oil physical refining exhibited very high levels of phenolic compounds, in most part represented by non-flavonoid compounds; therefore, they can be considered as a potential antioxidant source. In particular, the WW-A extract—obtained with pure ethanol (99.8%) as an extraction solution—showed the highest total phenolic amount.

### 3.2. Quality Parameters and Total Phenol Content of Enriched Control Olive Oils (COOs)

To verify the suitability of the proposed two prototypes of phenol-enriched oils (PE-A and PE-B), the total phenol content as well as the values of the general quality parameters (free acidity, peroxide value, and K_270_) were evaluated (Table 3) and compared with those obtained from the COO. 

As seen, the values of the quality parameters determined in both phenol-enriched oils were within the range that makes them edible. 

Furthermore, in the operating conditions adopted, the phenolic content significantly increased in both prototypes PE-A and PE-B compared with the COO and the values were assumed to appear close to those indicated for several extra-virgin olive oils labeled with protected geographical indication (PGI) [29].

In particular, the concentration of total phenols in the enriched oils was 4.2-fold greater than that determined in the starting COO when extract A (ethanol 99.8%) was used, while this value increased to reach 5.2 when extract B (ethanol:diethyl ether (1:2 *v/v*)) was used for the oil enrichment.

As shown in Table 3, the bitter index (BI, K_225_) was directly proportional to the total phenolic content. Additionally, all samples analyzed could be classified as non-bitter or almost imperceptibly bitter oils, as they showed a value of K_225_ ≤ 0.25, which was assumed as a reference point to indicate the appearance of some bitter taste by chemical analysis [26]. Further studies are needed to perform a proper panel test to confirm this evidence by sensory characterization.

### 3.3. Antioxidant Capacity of the Olive Oils (COO and Phenol-Enriched Oils) by ABTS Assay

**Figure 1:**
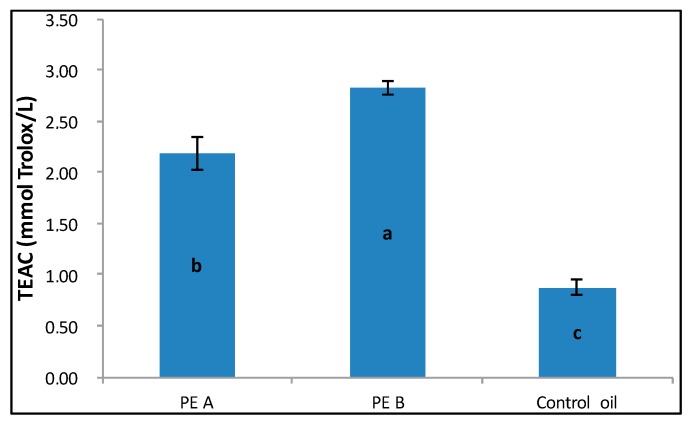
Trolox equivalent antioxidant capacity (TEAC) value determined for COO and both phenol-enriched olive oils. Parameters not sharing the same letter have a significantly different mean concentration (α = 0.05).

The antioxidant capacity of the oils was analyzed by ABTS assay before (COO) and after (PE-A and PE-B) the enrichment. As shown in Figure 1, the antioxidant capacity of both the enriched oils was significantly higher than that shown by the control oil. In particular, the highest value of TEAC was determined for PE-B oil, which was characterized by the highest concentration of phenolic compounds, represented by non-flavonoid fraction (see Table 3).

### 3.4. Polyphenols Recovery H_2_O_2_—Impaired Endothelial Cell Viability

**Figure 2:**
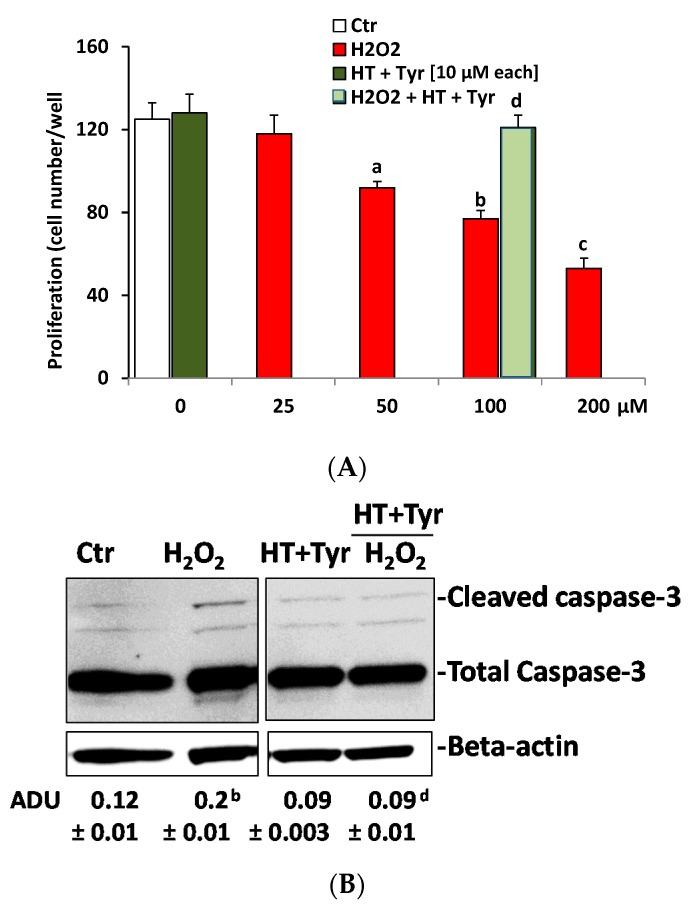
Polyphenols recover H_2_O_2_-induced HUVEC (human umbilical cord vein endothelial cells) proliferation and apoptosis. (**A**) HUVEC proliferation in response to H_2_O_2_ with/without HT + Tyr (48 h). Data are reported as cells/well ± SEM (*n* = 3 run in triplicate). Ctr = 1% FCS; ^a^ *p* < 0.05, ^b^ *p* < 0.01, ^c^ *p* < 0.001 versus Ctr, ^d^ *p* < 0.01 versus H_2_O_2_ 100 µM; (**B**) Cleaved caspase-3 in HUVEC exposed to H_2_O_2_ (100 µM) for 6 h, with/without HT + Tyr (10 µM each). Beta-actin was used for normalization. Total caspase-3 is shown as control of loading. Caspase-3 activity is expressed as arbitrary density unit (ADU). ^b^ *p* < 0.01 vs. 1% FCS, ^d^ *p* < 0.01 versus H_2_O_2_ 100 µM. The gels shown are representative of three runs with similar results.

The suspension of phenolic extracts in ethanol:diethyl ether (1:2 *v/v*) was not compatible with cell culture studies. Thus, for in vitro studies of phenolic extracts, the focus was on pure polyphenols. In both in vitro and in vivo studies, HT is the phenolic extract of olive oil that has been mainly investigated for its biological properties. HT has widely been considered to be beneficial for health, as it has been reported to prevent atherosclerosis and cancer, and to exhibit antimicrobial and anti-inflammatory activities [30,31,32]. Recently, it has been reported that HT inhibited colon cancer cell proliferation in vitro, and reduced tumor-mass growth in vivo in a mouse model [33]. Furthermore, it a possible mechanism has also been reported where HT increased the degradation of an epidermal growth factor receptor (an oncogenic signal in colon cancer) by promoting its ubiquitination [33]. Here, to investigate the properties of the polyphenols present in the phenolic extracts in a biological setting, the extracts were mimicked using a mix of HT and Tyr—the two major polyphenols of virgin olive oil [34]. The activity of the polyphenols was evaluated at concentrations between 0.1 and 100 µM in endothelial cell culture experiments, corresponding to nutritional/healthy-recommended polyphenol doses obtained from olive oil consumption for its anti-atherogenic activity [35]. First, it was investigated whether HT and Tyr would affect endothelial cell growth, per se and in combination. As shown in Table 4, HUVEC growth was unaffected by polyphenols at any concentration tested in the 48 h incubation. The effects exerted by the 100 µM concentration on cell viability were borderline toxic, and therefore in other experiments reported here, HT and Tyr were used at 10 µM, and served also for the mix. Next, the mix was tested for its effect on HUVEC growth, and as the combination of two compounds did not induce significant effects when compared with the control condition (1% FCS, Figure 2A), it was concluded that HT and Tyr—either per se or in combination (each at 10 µM)—did not affect endothelial viability.

Reactive oxygen species (ROS)—including hydrogen peroxide (H_2_O_2_) and superoxide radicals—have been reported as the cause of endothelial cell damage during atherosclerosis [30]. In particular, the endothelium has been reported to undergo apoptosis when exposed to ROS [36,37]. 

Thus, a combination of polyphenols was investigated for its effects on HUVEC growth when challenged with H_2_O_2_. Exposure of HUVEC to graded concentrations of H_2_O_2_ (25, 50, 100, and 200 µM) produced a significant decrease in cell count when compared to the control condition (1% FCS), demonstrating the sensitivity of HUVEC to ROS (Figure 2A). A selection of 100 µM H_2_O_2_ was made for further experiments since it inhibited cell viability by 60%. Co-incubation of HUVEC with the mix of polyphenols recovered endothelial cells from the damage produced by H_2_O_2_ (100 µM), indicating the protective effects of polyphenols on ROS-induced endothelium damage (Figure 2A). Given the reduction of cell number (suggestive of apoptosis produced by H_2_O_2_ treatment), we then measured the intracellular level of caspase-3 activity, long recognized as a reliable biochemical correlate of apoptotic events. Indeed, H_2_O_2_-induced apoptosis is known to be mediated through caspase-3 activation [31,36]. The results (shown in Figure 2B) demonstrated a significant increase of cleaved caspase-3 (the active form of caspase-3) in the HUVEC incubated with H_2_O_2_ for 6 h when compared with the control condition (1% FCS) (*p* < 0.01). The addition of the mix reversed the effect of H_2_O_2_ on caspase activity (*p* < 0.01) (Figure 2B). 

### 3.5. The Recovering Effects of Polyphenols are Linked to the Expression of Antioxidant Enzymes in Endothelium

**Figure 3:**
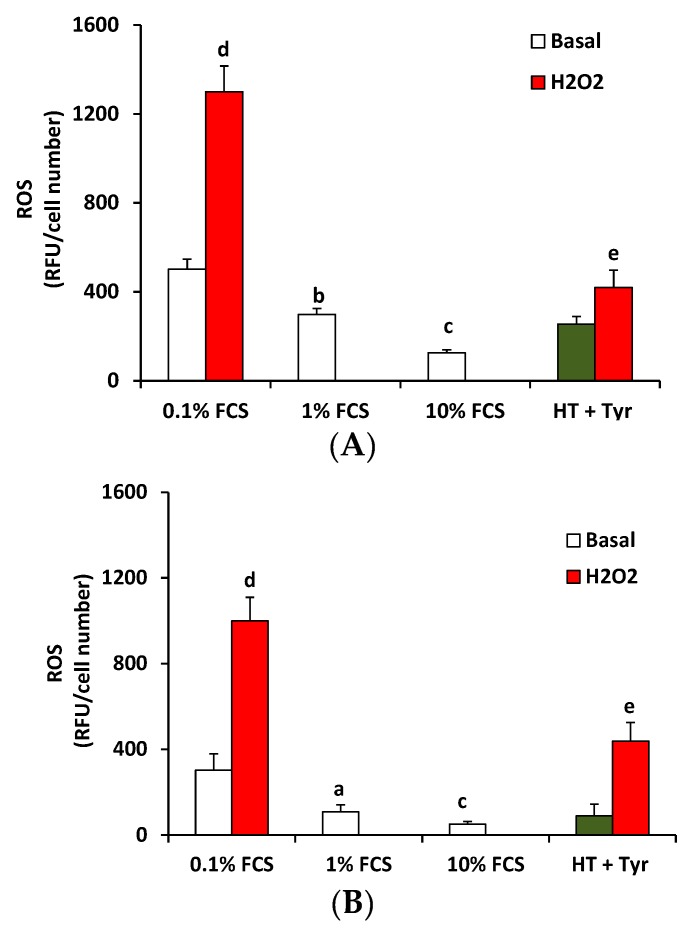
The HT + Tyr combination decreases reactive oxygen species (ROS) levels in HUVEC exposed to H_2_O_2_. HUVEC were stimulated with HT + Tyr (10 μM each, green bar) in 0.1% FCS for (**A**) 30 min or (**B**) 18 h and then exposed to H_2_O_2_ (100 μM, 90 min). Data are expressed as relative fluorescent units (RFU)/cell number (*n* = 3). ^a^ *p* < 0.05, ^b^ *p* < 0.01, ^c^ *p* < 0.001 vs. 0.1% FCS, ^d^ *p* < 0.001 vs. 0.1% FCS, ^e^ *p* < 0.001 vs. H_2_O_2_.

Next it was analyzed whether the recovering effects of polyphenols on HUVEC viability was linked to their direct antioxidant activity or to other biological effects. To verify this hypothesis, first ROS production was measured in HUVEC pre-treated with the mix of polyphenols for 30 min or 18 h and then stimulated with H_2_O_2_ (100 μM, in 0.1% FCS) for 90 min by monitoring the oxidation of intracellular 2′,7′-dichlorodihydrofluorescein diacetate (H2DCFDA). ROS production was increased in treated cells compared to either low serum condition (0.1% FCS), control condition (1% FCS), or optimal serum condition (10% FCS), and this effect was significantly reduced in HUVEC pre-treated with polyphenols, either for 30 min or for 18 h (Figure 3A,B), suggesting both a direct and indirect antioxidant activity of polyphenols.

**Figure 4:**
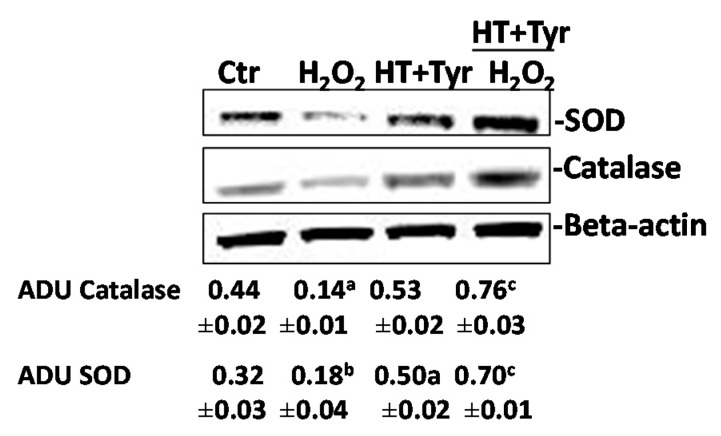
Combination of polyphenols rescues superoxide dismutase (SOD) and catalase expression in HUVEC exposed to H_2_O_2_. Western blot analysis of SOD and catalase in HUVEC exposed to H_2_O_2_ (100 µM) with/without HT + Tyr (10 µM each). Beta-actin is used for normalization. Expression of catalase or SOD is reported as arbitrary density unit (ADU). ^a^ *p* < 0.05, ^b^ *p* < 0.01 vs. 1% Ctr = FCS; ^c^ *p* < 0.01 versus H_2_O_2_. The gels are representative of three runs with similar results.

Next, it was investigated whether the polyphenols’ inhibitory activity on ROS levels at 18 h was mediated by the recovering levels of antioxidant-related signals (SOD and catalase) in endothelial cells. It was found that 100 μM H_2_O_2_ decreased SOD and catalase expression at 18 h (Figure 4). The mix of polyphenols (which had a mild activity per se) in combination with 100 μM H_2_O_2_ recovered SOD and catalase expression when compared with the control condition (1% FCS), suggesting that the long-lasting protective effects of polyphenols on cell damage induced by ROS might be linked to their indirect antioxidant properties.

Together, the in vitro findings showed that the mix of HT and Tyr significantly prevented the damage mediated by hydrogen peroxide, sustaining endothelial cell growth and the expression of antioxidant enzymes such as superoxide dismutase and catalase. Specifically, the combination of HT and Tyr prevented endothelial cells from entering the H_2_O_2_-induced apoptosis, restoring their viability and their inherent capacity to proliferate in response to serum (1% FCS). In the endothelial cells (known for their sensitivity to the external environment), the mechanism of the anti-apoptotic effect exerted by the mix HT plus Tyr appeared to be either dependent or independent of scavenging activity, and the mix of polyphenols provided a survival advantage capable of overriding the cytotoxic insult. In this context, HT and Tyr appeared to be alternative agents to the known purely scavenger molecules in reducing oxidative stress-induced vascular damage. The knowledge that these small molecules could prevent the persistent damaging effects of ROS might have implications for the design of novel therapies for cardiovascular pathologies where the dysfunction of the endothelium was the underlying causative factor. 

These findings, together with the effects of HT on cancer cells, may provide a rational mechanistic framework for the health benefits reported in epidemiological studies on the Mediterranean diet. 

## 4. Conclusions

At laboratory scale, different extraction solutions were tested to develop an extraction method to recover high added-value compounds from the wastewater produced during the physical refining of olive oil. The extracts were then evaluated for their potential application in the phenol-enrichment and stabilization of commercial olive oils. 

The results showed that wastewater collected during the physical refining process could be considered as a good source of bioactive compounds useful for a significant increase of nutraceutical value, as well as of the antioxidant capacity of olive oils. 

Currently, many studies on olive oil-mediated beneficial health effects have indicated that it reduced oxidation of the low-density lipoprotein carrying cholesterol (LDL-C), and inhibited thrombogenic events [38,39,40]. In agreement with these experimental observations, epidemiological and clinical studies have reported that olive oil reduced the incidence of cardiovascular diseases [41,42]. In this study, in a model of in vitro endothelial cells, the combination of HT with Tyr—the two major olive oil polyphenols which mimic the phenolic extracts from COO—when used at the recommended concentration from EVOO consumption, preserved cell functions from oxidative damage, rescuing their antioxidant properties. 

Further studies are needed to investigate the protective properties of phenolic extracts in enriched olive oil, as well as to validate the proposed method and to explore the feasibility of its industrial scale-up. In this context, close attention should be paid to the following main aspects: the evaluation of the chemical composition of the phenol-enriched olive oils (PE-A and PE-B); the determination of the possible residue of solvents in the industrial enriched oil; the setup and standardization of the industrial process; the evaluation of the cost effectiveness of the industrial production; and the quantification of the added value that could be assigned to the new product. 

## Figures and Tables

**Table 1:** Chemical composition control oil (COO) and wastewater (WW).

Parameter	Control Olive Oil (COO)	Wastewater (WW)
Free acidity (% of oleic acid)	0.12 ± 0.01	--
Peroxide value (meq O_2_/kg)	5.46 ± 0.03	--
K_270_	0.56 ± 0.03	--
pH	--	3.32 ± 0.02
Density	--	1.03 ± 0.01
Dry matter (d.m. %)	--	11.53 ± 0.10
Total phenol content (g/L of gallic acid)	0.025 ± 0.002	1.880 ± 0.004

**Table 2:** Total phenol content of wastewater and wastewater extracts.

Sample	Total Phenol Content (g/L) as Gallic Acid	% of Non-Flavonoid Phenols
Wastewater (WW)	1.880 a	92%
WW Extract A (WW-A)	0.430 b	--
WW Extract B (WW-B)	0.292 c	--

Parameters not sharing the same letter have a significantly different mean concentration (α = 0.05).

**Table 3:** Quality parameters and total phenol content of control oil and phenol-enriched prototypes.

Sample	Free Acidity (% of Oleic Acid)	Peroxide Value (meq O_2_/kg)	K_270_	ΔK	K_225_	Bitter Index (BI)	Total Phenol Content (g/kg of Gallic Acid)	% of Non-Flavonoid Phenols
COO	0.12 a	5.46 a	0.56 a	≤0.15	0.10 c	0.52 c	0.025 c	99.4
PE-A	0.12 a	5.47 a	0.57 a	≤0.15	0.15 b	1.20 b	0.105 b	74.9
PE-B	0.12 a	5.47 a	0.57 a	≤0.15	0.18 a	1.52 a	0.131 a	100

Parameters not sharing the same letter have a significantly different mean concentration (α = 0.05).

**Table 4:** Endothelial cell number in response to polyphenols alone or in combination during 48 h culture.

Polyphenol Concentration Tested	Ctr	HT	Tyr	HT + Tyr
0	121 ± 4	---	---	---
0.1 µM	---	130 ± 7	123 ± 6	---
1 µM	---	126 ± 5	124 ± 4	---
10 µM	---	127 ± 5	125 ± 8	130 ± 8
100 µM	---	112 ± 6	108 ± 10	---

Data are reported as cells/well ± SEM (*n* = 4 run in triplicate). Polyphenols did not affect the number of endothelial cells in culture. Ctr = 1% fetal calf serum (FCS). Ctr: control; HT: hydroxytyrosol; Tyr: tyrosol.
